# Association between enteral formula type and nutritional failure in critically ill children: A single-center retrospective study

**DOI:** 10.1007/s00431-026-06968-6

**Published:** 2026-05-05

**Authors:** Ayşe Aşık, Muhterem Duyu, Sinem Aydöner, Çiğdem Ulukaya Durakbaşa

**Affiliations:** 1 Department of Pediatric Intensive Care, Goztepe Prof. Dr. Suleyman Yalcin City Hospital, Istanbul, Türkiye; 2 Department of Pediatric Surgery, Goztepe Prof. Dr. Suleyman Yalcin City Hospital, Istanbul, Türkiye; 3 Department of Pediatric Surgery, Istanbul Medeniyet University, Istanbul, Türkiye

**Keywords:** Intensive care units, Pediatric, Enteral nutrition, Dietary proteins, Hydrolyzed, Dietary fiber, Feeding intolerance

## Abstract

Enteral nutrition is a fundamental component of care in critically ill children, yet uncertainty remains about the most appropriate formula. The aim of this study is to compare peptide-based formulas (PBF) and fiber-enriched polymeric formulas (FEF) with respect to feeding failure, intolerance, and progression toward nutritional goals in a PICU population. This study summarizes a single-center experience in a tertiary Pediatric Intensive Care Unit in Türkiye and included 225 children, aged 1–18 years, who received enteral nutrition for at least 48 h between 2021 and 2024. A total of 116 patients were started on PBF and 109 on FEF. Feeding failure was defined as discontinuation of enteral nutrition, switching formula, or failure to achieve ≥ 66% of prescribed caloric targets by day 7. Feeding intolerance rates were similar between groups. However, feeding failure occurred more frequently in the FEF group compared to the PBF group (43.1% vs. 29.3%, *p* = 0.037). After adjustment for relevant clinical variables, initiation with FEF remained independently associated with feeding failure (OR 2.18; 95% CI 1.22–3.88; *p* = 0.009). Nutritional risk scores improved earlier over the first 14 days in the PBF group. Patients receiving PBF also demonstrated higher protein delivery and reached caloric targets sooner.

* Conclusion:* Our results showed that peptide-based formula was associated with lower rates of feeding failure and faster achievement of nutritional targets. While causality cannot be inferred from this retrospective design, formula selection may represent an important, potentially modifiable factor in nutritional management during the PICU stay.

**Table:** 

**What is Known:** *• Enteral nutrition is a fundamental component of care for critically ill children, yet achieving optimal gastrointestinal tolerance and meeting nutritional goals remains a significant clinical challenge. * *• Although specialized enteral formulas are developed to improve feeding tolerance, evidence directly comparing the efficacy of peptide-based versus fiber-enriched polymeric formulas in pediatric intensive care units is limited.* **What is New:** *• Initiation of enteral nutrition with a peptide-based formula is associated with a significantly lower rate of feeding failure compared to fiber-enriched polymeric formulas in critically ill children.* *• Patients receiving peptide-based formulas demonstrate earlier improvement in nutritional risk scores and achieve their prescribed caloric and protein targets more rapidly.*

**Supplementary Information:**

The online version contains supplementary material available at 10.1007/s00431-026-06968-6.

## Introduction

Enteral nutrition is a crucial therapeutic intervention for critically ill children, as it maintains gastrointestinal integrity, modulates the inflammatory response, and ensures the timely delivery of energy and protein during periods of increased metabolic stress [1, 2]. Children admitted to the Pediatric Intensive Care Unit (PICU) face a high risk of nutritional deterioration due to the combined effects of systemic inflammation, metabolic stress, and disruption of catabolic balance [3, 4]. While enteral nutrition (EN) is the cornerstone of nutritional support for these children, signs of EN intolerance, such as vomiting, diarrhea, and abdominal distension, have been reported in up to 57.1% of patients [5]. Advances in enteral nutrition have enabled the development of specialized formulas designed to improve nutrient delivery and gastrointestinal tolerance by modifying protein structure, lipid composition, caloric density, and fiber content. Despite these advances, achieving and maintaining effective enteral nutrition in critically ill pediatric patients remains a significant clinical challenge. Interruption of enteral feeding or inability to sustain adequate energy and protein delivery may lead to nutrition-related complications, including cumulative macronutrient deficits, delayed achievement of nutritional targets, recurrent feeding interruptions, and increased reliance on parenteral nutrition, thereby adversely affecting nutrition-related outcomes in critically ill children [1, 6–8].

To enhance feeding tolerance, specialized enteral formulations with distinct compositional characteristics have been developed [Supplementary Fig.1. Peptide-based formulas (PBFs) have been proposed to improve tolerance by facilitating faster gastric emptying [9, 10], whereas fiber-enriched polymeric formulas (FEFs) are thought to confer benefits through modulation of intestinal motility and support of the gut microbiota [1, 11]. However, evidence directly comparing these two nutritional strategies with respect to feeding tolerance, enteral feeding failure, and clinical outcomes in critically ill pediatric and adult populations remains limited and inconsistent [12–15]. Therefore, this study primarily aimed to compare PBFs and FEFs with respect to enteral feeding intolerance and feeding failure in critically ill children. Secondary objectives were to evaluate the effects of enteral formulas on enteral nutrition tolerance and continuity, nutritional adequacy and efficiency, and longitudinal nutritional risk assessment.

## Materials and methods

This was a single center, retrospective observational cohort study conducted in the tertiary Pediatric Intensive Care Unit of Göztepe Prof. Dr. Suleyman Yalcin City Hospital. The study was approved by the local Institutional Review Board (IRB approval no: 998; date: October 24, 2024) and conducted in accordance with the Declaration of Helsinki. Due to the study's retrospective nature using anonymized data, informed consent was waived by IRB. The study followed STROBE guidelines for observational studies [16].

All patients admitted to the PICU between October 2021 and October 2024 were screened for eligibility. Patients were included if they were aged 1–18 years, had a PICU length of stay longer than 72 h, received EN for at least 48 h via tube feeding (nasogastric, orogastric, gastrostomy or post pyloric), and were initiated on either a peptide-based formula or a fiber-enriched formula as the initial EN on regimen. Patients were excluded if total parenteral nutrition was initiated as the primary feeding method during PICU admission, if medical records were incomplete, or if enteral formulas other than the two predefined study formulas were used. For patients with more than one PICU admission during the study period, only the first admission was included in the analysis [Supplementary Fig. 2].

Given the retrospective nature of the study, the initial enteral formula was selected by the treating clinician and dietitian based on patient-specific clinical factors, and the unit's nutritional protocol, also considering the stock availability in the hospital pharmacy. Generally, PBFs were initiated in patients with anticipated gastrointestinal dysfunction, while FEFs were selected for those expected to tolerate standard enteral nutrition. However, the final decision remained at the discretion of the treating clinician.

Baseline demographic and clinical variables were recorded to characterize the study population and to account for potential confounding. These included age, sex, anthropometric measures and corresponding z-scores (when available) [17], primary reason for PICU admission, and presence of underlying comorbidities. Admission diagnoses were classified by organ system. Disease severity was assessed using validated pediatric scores, with The Pediatric Risk of Mortality score (PRISM III) [18] recorded at admission and the highest Pediatric Logistic Organ Dysfunction (PELOD) [19] score during follow-up used for analysis. Data on organ support and adjunctive therapies during PICU stay, including mechanical ventilation, vasoactive medications, and renal replacement therapy, were collected. Laboratory parameters relevant to nutritional and inflammatory status and metabolic abnormalities occurring during PICU stay were also recorded and considered potential confounders in analyses evaluating the association between enteral formula type and nutritional and clinical outcomes.

Feeding intolerance was assessed in accordance with the unit’s standardized nutritional monitoring protocol, which includes clinical signs such as nausea, vomiting, diarrhea, constipation, high GRV, aspiration pneumonia, gastrointestinal bleeding, and abdominal distension [Supplementary Nutrition Assessment and Monitoring Form].

The primary outcome was enteral feeding intolerance and feeding failure. Enteral feeding failure was defined as discontinuation of EN due to feeding intolerance related reasons; requirement for change of the initially selected enteral formula and/or transition to total parenteral nutrition; and/or failure to achieve at least 66% of the prescribed energy target by day 7 of EN [2]. Secondary outcomes included nutritional adequacy and efficiency, time to achievement of predefined energy target, and longitudinal nutritional risk. EN intolerance was assessed in accordance with the unit’s standardized nutritional monitoring protocol [Supplementary Nutrition Assessment and Monitoring Form]. Nutritional adequacy and efficiency outcomes included time to first achievement of predefined caloric targets, daily energy and protein intake during PICU stay, and the proportion of patients achieving at least 66% of prescribed energy targets by day 7. Longitudinal nutritional risk was evaluated using repeated Pediatric Yorkhill Malnutrition Score (PYMS) classifications during PICU stay [20]. All nutritional practices, monitoring, and data collection followed the institutional EN protocol detailed in the Supplemental Digital Content.

### Statistical analysis

As this study had a retrospective observational design, no formal sample size or power calculation was performed; the study cohort consisted of all patients admitted to the PICU during the study period who met the predefined inclusion criteria. Continuous variables were summarized as medians with interquartile ranges (IQR), and categorical variables were expressed as frequencies and percentages. Nutritional adequacy was evaluated using predefined clinically meaningful thresholds, including achievement of at least 66% of the prescribed energy target by day 7 of EN. Time to achievement of caloric targets was analyzed using Kaplan–Meier methods and compared between groups using the log-rank test.

To identify factors associated with enteral feeding failure, variables with a *p*-value < 0.1 in bivariate analyses and those considered clinically relevant based on prior literature were included in univariable logistic regression models [2, 3, 7]. Results were reported as adjusted odds ratios (aORs) with 95% confidence intervals (CIs). Longitudinal repeated measurements obtained during the PICU stay were analyzed using generalized estimating equations (GEE) to account for within-subject correlations. Patients with missing data required for specific analyses were excluded from those analyses, and a complete-case approach was applied. All statistical analyses were performed using IBM SPSS Statistics software (Version 26.0), and a two-sided *p*-value < 0.05 was considered statistically significant. Detailed information on statistical analyses are provided in the Supplemental Digital Content.

## Results

A total of 225 critically ill children receiving enteral nutrition were included, with 116 patients receiving a PBF and 109 receiving a FEF [Table 1]. Baseline demographic characteristics, disease severity scores (PRISM III and PELOD), primary diagnosis categories, and admission laboratory parameters were comparable between groups, with no statistically significant differences observed across baseline variables (all *p* > 0.05).

**Table 1:** Baseline demographic, clinical, nutritional, and laboratory characteristics of the study population and clinical outcomes during PICU stay

Variable		Total (n=225)	Peptide-based formula (n= 116)	Fiber-enriched polymeric formula (n=109)	*p* value
Age, months, median (IQR)		103 (65–129)	95 (70–119)	111 (60–135)	0.219
Sex, male, n (%)		119 (52.9)	59 (50.9)	60(55)	0.530
Weight, kg, median (IQR)		17.7 (11.2–24.11)	17.4 (11.7–23.1)	18.3 (11–25.4)	0.448
Height, cm, median (IQR)		114 (98–124)	112 (98–123)	115 (95–125)	0.422
BMI Z-score, kg/m^2^, median (IQR)		−0.7 (−3.1-0.3)	−0.7 (−2.7-0.2)	−0.8 (−3.1-0.5)	0.973
Primary diagnosis category, n (%)	Respiratory	69 (30.7)	30 (25.9)	39 (35.8)	0.114
	Neurologic	24 (10.7)	16 (13.8)	8 (7.3)	0.177
	Trauma	38 (16.9)	22 (19)	16 (14.7)	0.497
	Sepsis/infection	28 (12.4)	12 (10.3)	16 (14.7)	0.434
	Cardiac	24 (10.7)	12 (10.3)	12 (11)	>0.99
	Postoperative	26 (11.6)	12 (10.3)	14 (12.8)	0.706
	Other	14 (6.2)	10 (8.6)	4 (3.7)	0.208
Comorbidity present, n (%)		97 (43.1)	56 (48.3)	41 (37.6)	0.107
PRISM III score, median (IQR)		13 (7–21.5.5)	12.5 (8–18.8)	13 (4–23)	0.599
PELOD score (max), median (IQR)		16 (14–18)	15 (13–18)	16 (14–18)	0.351
Day 1	Albumin, g/dL, median (IQR)	3.2 (2.9–3.4)	3.2 (2.8–3.4)	3.2 (2.9–3.5)	0.570
	Sodium, mmol/L, median (IQR)	138 (136–139)	137 (136–139)	138 (135–140)	0.575
	Potassium, mmol/L, median (IQR)	4.3 (4–4.5)	4.3 (4.1–4.5)	4.3 (3.9–4.6)	0.623
	Phosphate, mg/dL, median (IQR)	3.6 (3.3–3.9)	3.6 (3.3–4.3)	3.5 (3.3–3.8)	0.202
	Urea, mg/dL, median (IQR)	25 (19–30)	24 (19–30)	25 (20–31)	0.235
	Glucose mg/dL, median (IQR)	110 (97–121)	111 (100–124)	109 (96–119)	0.199
Day 7	Albumin, g/dL, median (IQR)	3.1 (2.8–3.3)	3.1 (2.8–3.4)	3.1 (2.7–3.3)	0.861
	Sodium, mmol/L, median (IQR)	138 (136–141)	138 (136–140)	138 (136–142)	0.443
	Potassium, mmol/L, median (IQR)	4.4 (3.9–4.9)	4.4 (4–4.7)	4.4 (3.9–4.8)	0.781
	Phosphate, mg/dL, median (IQR)	3.8 (3.4–4.1)	3.8 (3.4–4.2)	3.8 (3.5–4.5)	0.488
	Urea, mg/dL, median (IQR)	22 (15–27)	21 (15–26)	22 (15–27)	0.201
	Glucose mg/dL, median (IQR)	100 (86–113)	101 (85–118)	96 (87–110)	0.209
IMV, n (%)		73 (32.4)	36 (31)	37 (33.9)	0.641
Duration of IMV, days, median (IQR)		4 (3–6.5)	5 (3–6.8)	4 (3–6.5)	0.872
Renal replacement therapy, n (%)		13 (5.8)	9 (7.8)	4 (3.7)	0.304
Plasmapheresis, n (%)		9 (4)	2 (1.7)	7 (6.4)	0.094
ECMO, n (%)		3 (1.3)	1 (0.9)	2 (1.8)	0.612
Neuromuscular blocking agents, n (%)		18 (8)	10 (4.4)	8 (3.6)	0.914
Gastric protective agents, n (%)		173 (76.9)	88 (75.9)	85 (78)	0.753
Use of prokinetic agents, n (%)		42 (18.6)	18 (15.5)	24 (22)	0.280
Sedative-Analgesic agents, n (%)		185 (82.2)	92 (79.3)	93 (85.3)	0.315
Vasoactive support, n (%)		28 (12.4)	11 (9.5)	17 (15.6)	0.235
PICU length of stay, days, median (IQR)		9 (6–11)	9 (5.3–14.3)	8 (6–11)	0.662
Mortality, n (%)		18 (8)	10 (8.6)	8 (7.3)	0.914

*BMI* body mass index, *PRISM III* Pediatric Risk of Mortality III score, *PELOD* Pediatric Logistic Organ Dysfunction score, *IMV* invasive mechanical ventilation, *ECMO* extracorporeal membrane oxygenation, *PICU* pediatric intensive care unit, *IQR* interquartile range

Enteral feeding practices and nutritional adequacy are summarized in Table 2. The route of enteral feeding, nutritional risk at admission, and time to enteral nutrition initiation were similar between groups. Daily enteral energy and protein intake on day 3 did not differ. By day 7, patients receiving the PBF achieved higher daily protein intake (median [IQR]: 1.7 [1.3–2.2] vs 1.6 [1.2–2.0] g/kg/day; *p* = 0.037), while energy intake remained comparable. Feeding failure was more frequently observed in the FEF group (43.1% vs 29.3%; *p* = 0.037). A higher proportion of patients in the peptide-based group remained on enteral nutrition on day 7, although this did not reach statistical significance (90.5% vs 80.7%; *p* = 0.055). Requirements for organ support and overall clinical outcomes, including PICU length of stay and mortality, did not differ between formula groups.

**Table 2:** Enteral feeding practices and nutritional adequacy, enteral tolerance, safety

Variable	Peptide-based formula (n=116)	Fiber-enriched polymeric formula (n=109)	p value
Enteral feeding route, n (%)			
Nasogastric	87 (75)	85 (78)	0.700
Orogastric	5 (4.3)	6 (5.5)	
Gastrostomy	22 (19)	15 (13.8)	
Post pyloric	2 (1.7)	3 (2.8)	
Pediatric Yorkhill Malnutrition Score, median (IQR)	2 (1–3)	2 (1–3)	0.431
Time to enteral nutrition initiation, days, median (IQR)	1.8 (1–2.5)	1.5 (1–2.5)	0.296
Enteral feeding continuation			
Enteral nutrition ongoing on day 3, n (%)	86 (74.1)	77 (70.6)	0.654
Enteral nutrition ongoing on day 7, n (%)	105 (90.5)	88 (80.7)	0.055
Daily delivered enteral intake			
Daily enteral energy intake on day 3, kcal/kg/day, median (IQR)	33 (25–46)	34 (25–42)	0.842
Daily enteral protein intake on day 3, g/kg/day, median (IQR)	1 (0.8–1.4)	1 (0.7–1.3)	0.396
Daily enteral energy intake on day 7, kcal/kg/day, median (IQR)	56 (40–69)	53 (41–66)	0.555
Daily enteral protein intake on day 7, g/kg/day, median (IQR)	1.7 (1.3–2.2)	1.6 (1.2–2.2)	**0.037**
Achieved ≥66 % of energy target by day 7, n (%)	89 (76.7)	77 (70.6)	0.363
Feeding interruptions and adjuncts			
Number of days with enteral feeding interruption, median (IQR)	0.8 (0.2–2.2)	1 (0–2)	0.949
Safety and complications during PICU stay			
Development of metabolic abnormalities, n (%)	19 (16.4)	16 (14.7)	0.867
Refeeding syndrome, n (%)	2 (1.7)	3 (2.8)	0.675
Enteral tolerance outcomes			
Feeding intolerance, n (%) *	42 (36.2)	45 (41.3)	0.434
• Diarrhea, n (%)	24 (20.7)	16 (14.7)	0.315
• Nausea/Vomiting, n (%)	14 (12.1)	17 (15.6)	0.566
• Abdominal distension, n (%)	11 (9.5)	19 (17.4)	0.120
• Constipation, n (%)	15 (12.9)	10 (9.2)	0.494
• High GRV/feeding hold***, n (%)	10 (8.6)	16 (14.7)	0.226
• Aspiration pneumonia, n (%)	3 (2.6)	5 (2.2)	0.488
• Gastrointestinal bleeding, n (%)	2 (1.7)	3 (2.8)	0.675
Feeding failure, n (%)	34 (29.3)	47 (43.1)	**0.037**
• Formula changes, n (%)	7 (6)	13 (11.9)	0.188
• Transition to parenteral nutrition, n (%)	8 (6.9)	10 (9.2)	0.701
• Inadequate energy intake (<66 % of target) on day 7, n (%)	27 (23.3)	32 (29.3)	0.363
– Feeding intolerance related, n (%)	19 (16.4)	24 (22)	0.365
– Non feeding related **, n (%)	8 (6.9)	8 (7.3)	>0.99

GRV, gastric residual volume; IQR, interquartile range

* Included diarrhea, vomiting, abdominal distension, constipation, high gastric residual volume/feeding hold, aspiration pneumonia, and gastrointestinal bleeding. More than one feeding intolerance may be observed in a single patient

** Interruptions due to procedures, hemodynamic instability, or airway-related issues

*** Temporary interruption of enteral nutrition lasting ≤ 24 h

When stratified by feeding failure status, patients with feeding failure were younger (median [IQR]: 88.0 [53.0–124.0] vs 106.5 [74.8–138.0] months; *p* = 0.020), had a higher prevalence of comorbidities (54.3% vs 36.8%; *p* = 0.016), and higher PRISM III scores (median [IQR]: 14.0 [10.0–24.0] vs 12.0 [6.0–18.0]; *p* = 0.008). Feeding failure was also more frequently observed among patients receiving a FEF (58.0% vs 42.0%; *p* = 0.044) [Supplementary Table 1].

Feeding failure occurred in 81 of 225 patients (36.0%), providing 81 outcome events for regression modeling [Table 3]. Variables meeting screening criterion (*p* < 0.10) were evaluated in logistic regression. In univariable analysis, comorbidity (OR 2.04; 95% CI, 1.17–3.55; *p* = 0.011), fiber-enriched formula use (OR 1.83; 95% CI, 1.05–3.17; *p* = 0.032), and younger age (OR 0.99 per month; 95% CI, 0.988–1.000; *p* = 0.072) were associated with higher feeding failure odds, while weight (OR 0.98 per kg; 95% CI, 0.947–1.013; *p* = 0.221) and PRISM III score (OR 1.02 per point; 95% CI, 0.995–1.049; *p* = 0.107) were not. Variables with *p* < 0.10 in univariable analyses were included in multivariable logistic regression. In the multivariable model, comorbidity (adjusted OR 2.30; 95% CI, 1.29–4.09; *p* = 0.005) and FEF use (adjusted OR 2.18; 95% CI, 1.22–3.88; *p* = 0.009) remained independently associated with feeding failure, and age retained a modest association (adjusted OR 0.99 per month; 95% CI, 0.987–1.000; *p* = 0.042); weight and PRISM III score were not independently associated with feeding failure.

**Table 3:** Independent predictors of enteral feeding failure in critically ill children

	Univariate analysis			Multivariate analysis		
	OR	%95 CI	*p*	OR	%95 CI	*p*
Age (per month)	0.99	0.988–1.000	**0.072**	0.99	0.987–1.000	**0.042**
Weight (per kg)	0.98	0.947–1.013	0.221			
Presence of comorbidity	2.042	1.174–3.550	**0.011**	2.301	1.294–4.094	**0.005**
PRISM III score (per point)	1.022	0.995–1.049	0.107			
Fiber-enriched polymeric formula (reference: peptide-based formula)	1.828	1.054–3.172	**0.032**	2.175	1.219–3.882	**0.009**

*OR* odds ratio, *CI* confidence interval,  *PRISM III* Pediatric Risk of Mortality III score

**Fig. 1:**
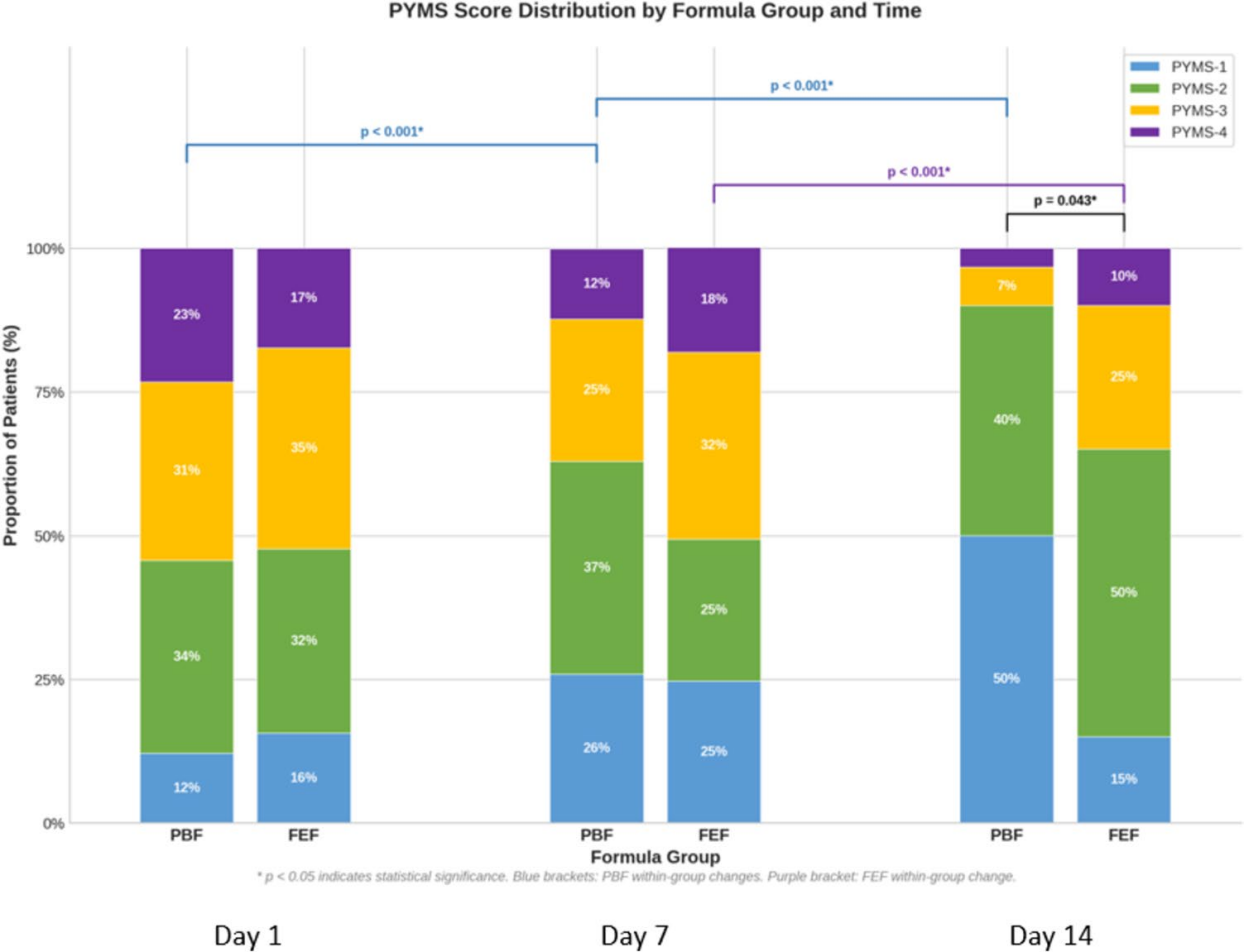
Stacked bar chart shows PYMS score classifications on days 0, 7, and 14 for patients receiving Peptide-based Formula (PBF) versus Fiber-enriched polymeric formula (FEF). Comparisons between PBF and FEF showed no significant differences on day 0 and day 7 (*p* = 0.629, *p* = 0.279), but showed significance on day 14 (*p* = 0.043). Within-group analyses showed significant changes in PYMS categories in the PBF group from day 0 to 7 and day 7 to 14 (*p* < 0.001). The FEF group showed no change between day 0 and 7 (*p* = 0.379), but significant change from day 7 to 14 (*p* < 0.001). Longitudinal analysis of nutritional risk scores, available for *n* = 225 at day 0, *n* = 158 at day 7, and *n* = 50 at day 14, showed earlier improvement in the PBF group

Temporal patterns of nutritional response favored the PBF group. Kaplan–Meier analysis showed earlier achievement of caloric targets in the PBF group versus FEF group (mean 5.85 days; 95% CI, 5.50–6.20 vs 6.44 days; 95% CI, 6.13–6.75) [Supplementary Fig. 3]. While median time was 6 days for both groups, PBF showed faster progression (log-rank test, *p* = 0.035). Longitudinal analysis of nutritional risk scores showed earlier improvement in the PBF group [Fig. 1]. PYMS distribution in PBF shifted significantly from Day 0 to 7 and Day 7 to 14 (both *p* < 0.001). The FEF group showed no change between Day 0 and 7 (*p* = 0.379), but shifted significantly between Day 7 and 14 (*p* < 0.001). Both groups shifted toward lower PYMS categories over time, with increased groups 1–2 and decreased groups 3–4; this transition occurred earlier in PBF. No between-group differences existed at admission or Day 7 (*p* = 0.629, *p* = 0.279), but differences emerged by Day 14 (*p* = 0.043).

Longitudinal changes in nutritional outcomes during PICU stay were evaluated using generalized estimating equation (GEE) models [Table 4]. Daily enteral energy intake increased significantly over time, with a mean increase of 3.50 kcal/kg/day per day (β = 3.50; 95% CI, 3.06–3.93; *p* < 0.001), indicating progressive advancement toward caloric targets irrespective of formula type. Energy intake showed no difference between formula groups at baseline and time by formula interaction [(β =  − 0.98; 95% CI, − 4.47 to 2.52; *p* = 0.583), (β = 0.58; 95% CI, − 0.09 to 1.24; *p* = 0.088) respectively]. Daily enteral protein intake increased over time (β = 0.108 g/kg/day per day; 95% CI, 0.092–0.123; *p* < 0.001), with greater increase in PBF versus FEF group (interaction β = 0.036 g/kg/day; 95% CI, 0.012–0.059; *p* = 0.003). Patients receiving PBF showed faster protein delivery escalation during observation. No significant longitudinal differences were observed between formula groups in ongoing enteral nutrition or high GRV/related feeding holds (ongoing enteral nutrition: OR 0.97; 95% CI, 0.84–1.13; *p* = 0.726; high GRV/feeding hold: OR 0.77; 95% CI, 0.30–2.02; *p* = 0.598).

**Table 4:** Longitudinal changes in nutritional outcomes according to enteral formula type analyzed using generalized estimating equations*

Outcome	Effect	Estimate	%95 CI	*p* value
Daily energy intake per kg (kcal/kg/day)	Day	β = 3.50	3.06–3.93	** < 0.001**
	Formula Group (PBF vs FEF)	β = − 0.98	− 4.47 to 2.52	0.583
	**Interaction between time and formula group**	β = 0.58	− 0.09 to 1.24	0.088
Daily protein intake (g/kg/day)	Day	β = 0.108	0.092–0.123	** < 0.001**
	Formula Group (PBF vs FEF)	β = − 0.035	− 0.142 to 0.072	0.519
	**Interaction between time and formula group**	β = 0.036	0.012–0.059	**0.003**
Enteral nutrition continuation (daily)	Day	OR = 0.76	0.70–0.84	** < 0.001**
	Formula Group (PBF vs FEF)	OR = 0.97	0.60–1.57	0.892
	**Interaction between time and formula group**	OR = 0.97	0.84–1.13	0.726
High gastric residual volume or feeding hold (daily)	Day	OR = 0.73	0.51–1.06	0.102
	Formula Group (PBF vs FEF)	OR = 1.05	0.12–9.28	0.966
	**Interaction between time and formula group**	OR = 0.77	0.30–2.02	0.598

*PBF*, peptide-based formula; *FEF*, fiber-enriched polymeric formula; *CI*, confidence interval; *OR*, odds ratio; *GRV*, gastric residual volume.

* Values are presented as β-coefficients for continuous outcomes and odds ratios (OR) for binary outcomes, each with corresponding 95% confidence intervals (CI), derived from generalized estimating equation models. The interaction term (Day × Formula Group) represents the difference in temporal trends between formula groups.

## Discussion

In this retrospective cohort of critically ill children receiving EN, formula type was associated with feeding tolerance, feeding failure and nutritional efficiency. With respect to the primary outcome, initiation of enteral nutrition with a PBF was independently associated with a lower risk of feeding failure compared with FEF. As a key secondary outcome, patients receiving a PBF achieved higher protein delivery by day 7 and exhibited a more pronounced longitudinal increase in daily protein intake over time; in contrast, advancement in energy delivery was primarily related to the passage of time rather than the type of enteral formula used. In addition, time-to-event and longitudinal visualizations demonstrated earlier achievement of caloric targets and more rapid improvement in nutritional risk profiles among patients receiving peptide-based feeding, indicating a consistent temporal pattern favoring PBF in terms of nutritional adequacy and tolerance during PICU stay.

Given the heterogeneity and inconsistent definitions of individual intolerance symptoms across studies, feeding intolerance has been reported in 20.0% to 69% of critically ill pediatric patients, highlighting the challenges of sustaining EN in this population [21]. In our cohort, clinical signs of feeding intolerance were observed in 38.6% of patients. Feeding intolerance is clinically relevant because it may lead to interruption or discontinuation of EN, necessitate changes in the initially selected enteral formula or transition to parenteral nutrition, and ultimately result in failure to achieve prescribed nutritional energy targets. For this reason, to capture the spectrum of clinically relevant adverse consequences associated with feeding intolerance, we selected enteral feeding failure, as operationally defined in this study, as the primary outcome. This outcome reflects the inability to maintain EN over time while accounting for both gastrointestinal tolerance and nutritional adequacy. Feeding failure, occurring in 29.3% of patients, is a more robust endpoint than documented intolerance symptoms in retrospective cohort studies.

PBFs are commonly preferred in patients with gastrointestinal intolerance because their hydrolyzed protein structure and lipid composition facilitate nutrient absorption and may improve tolerance during symptoms [8, 9, 26, 29]. FEFs have been developed to support gastrointestinal function by improving intestinal motility, stool consistency, and gut microbiota composition, with the aim of reducing gastrointestinal intolerance, particularly diarrhea, during prolonged enteral feeding [31]. However, clinical evidence supporting a consistent benefit of FEFs on feeding tolerance in critically ill pediatric patients remains limited. In our cohort, although the frequency of individual gastrointestinal intolerance symptoms did not differ significantly between formula groups, feeding failure occurred less frequently among patients receiving PBF.

Reported feeding intolerance rates with PBFs in PICU studies vary widely depending on outcome definitions, ranging approximately from 11 to 69% [3, 21, 22], while rates reported in adult intensive care unit populations range approximately from 9 to 30% [24–28]. Pediatric trials comparing PBFs with other enteral formulations have yielded inconsistent findings. One randomized controlled trial favored standard polymeric formulas based on lower feeding intolerance [29], whereas another reported no significant difference in feeding intolerance rates between formula types [22]. In contrast, adult retrospective studies have more consistently reported lower feeding intolerance with PBFs [23, 25]. In our cohort, feeding intolerance rates were comparable between PBF and FEF groups (36.2% vs 41.3%), whereas feeding failure occurred significantly less frequently in the PBF group (29.3% vs 43.1). This finding supports a potential advantage of PBFs in sustaining enteral nutrition beyond symptom based intolerance. Notably, unlike most prior studies comparing PBFs with standard polymeric formulas, our analysis specifically focused on FEFs.

Adequate early energy and protein intake reduces nutritional interruptions and cumulative macronutrient deficits in critically ill patients [32–36]. Achieving more than 2/3 of targeted energy and protein intake within the first week has been associated with improved clinical outcomes [37, 38]. Beyond reducing feeding failure, our study showed PBF use led to more efficient nutritional targets. In our cohort, PBFs were associated with a more favorable temporal pattern of nutritional efficiency. Compared with FEFs, PBFs facilitated earlier achievement of caloric goals and a more rapid escalation of protein delivery during the early phase of critical illness, supporting the concept that PBFs may enhance the continuity and sustainability of enteral nutrition in critically ill children. The observed higher protein intake in the PBF group, while energy intake remained similar, likely reflects a combination of two factors: the higher protein to energy ratio inherent to the peptide-based formula and the lower rate of feeding failure, which allowed for more consistent enteral delivery over time. We acknowledge that if both formulas had identical protein concentrations, the magnitude of the observed difference in protein intake would likely have been smaller, and the independent contribution of feeding tolerance to protein delivery would have been more clearly delineated. Therefore, the higher protein intake in the PBF group should not be interpreted as solely attributable to improved tolerance, but rather as a combined effect of formula composition and feeding continuity.

Our study builds on the existing literature by framing malnutrition risk in the PICU not as a fixed characteristic determined at admission, but as a dynamic condition that may change in response to the clinical course of critical illness. Although most studies focus on malnutrition at admission or single time point screening using tools such as the PYMS [19], emerging evidence indicates that nutritional risk in the PICU evolves with the clinical course, underscoring the need for repeated assessments to identify hospital-acquired malnutrition and improve risk stratification [39, 40]. While an overall improvement in malnutrition risk profiles over time was observed in both groups, the use of repeated and formula-specific nutritional assessments enabled a more sensitive evaluation of how formula selection and subsequent formula changes influenced malnutrition risk trajectories. Through this dynamic approach, we were able to demonstrate an earlier and more pronounced improvement in malnutrition risk score within the first week among patients receiving PBFs, highlighting the clinical relevance of continuous nutritional risk monitoring when evaluating enteral feeding strategies.

This study has several limitations. Its single-center, retrospective design may limit generalizability, and formula selection was not randomized, introducing potential confounding by indication. In addition, heterogeneity in outcome definitions limits direct comparison with prior studies. Finally, follow-up was limited to the PICU stay, and long-term nutritional outcomes were not assessed. Moreover, the difference in protein concentration between the two formula types represents a compositional confounder that limits the ability to isolate the independent effect of feeding tolerance on protein delivery. Future studies using formulas with matched macronutrient profiles would be needed to disentangle the contributions of formula composition and tolerance to nutritional outcomes. Furthermore, formula selection was based on clinical judgment rather than randomization, introducing potential confounding by indication. Although we adjusted for key clinical variables, unmeasured confounders in formula selection may have influenced the observed associations.

Enteral nutrition is a fundamental component of care in critically ill children. Our findings suggest that, in a setting where evidence-based guidance on optimal formula selection remains limited, the choice of enteral formula may represent a clinically relevant and potentially modifiable factor influencing nutrition-related outcomes. Although feeding intolerance rates did not differ significantly between formula types, PBFs was associated with a lower risk of feeding failure and more favorable secondary nutritional outcomes, including earlier achievement of caloric goals, faster protein delivery escalation, and earlier improvement in nutritional risk profiles assessed by serial PYMS measurements. These findings suggest that enteral formula selection represents a modifiable factor influencing the sustainability and adequacy of enteral nutrition in the PICU. The composite definition of feeding failure used in this study may offer a pragmatic and clinically meaningful outcome for future research aimed at improving the assessment and standardization of nutrition-related outcomes in pediatric critical care.

## Supplementary Information

Below is the link to the electronic supplementary material.

ESM 1 (DOCX 410 KB)

## Data Availability

The datasets generated and/or analyzed during the current study are not publicly available due to institutional and patient confidentiality restrictions but are available from the corresponding author on reasonable request and with institutional approval.
