# Supplementary material for: Association between enteral formula type and nutritional failure in critically ill children: A single-center retrospective study
Source: Eur J Pediatr. 2026 May 5;185(6):346. doi: 10.1007/s00431-026-06968-6 (PMC13139206; doi:10.1007/s00431-026-06968-6)
Supplement: Supplementary file 1 — (DOCX 410 KB) [file 431_2026_6968_MOESM1_ESM.docx]

## **SUPPLEMENTARY MATERIAL**

**TABLE OF CONTENTS**

[**SUPPLEMENTARY MATERIAL** 1](#_Toc227396717)

[**Supplementary Figure 1. Nutritional composition of the enteral formulas used in the study (per 100 mL)** 2](#_Toc227396718)

[**Supplementary Figure 2. Study flow diagram and patient selection process** 3](#_Toc227396719)

[**Supplementary Enteral Nutrition and Nutritional Monitoring Algorithm** 4](#_Toc227396720)

[**Supplementary PICU Nutrition Assessment and Monitoring Form** 12](#_Toc227396721)

[**Supplementary Statistical Methods** 18](#_Toc227396722)

[**Supplementary Figure 3. Kaplan–Meier Analysis of Time to Caloric Goal Achievement by Enteral Formula Type** 22](#_Toc227396723)

[**Supplementary Table 1: Feeding Failure Comparison Table** 23](#_Toc227396724)

# **Supplementary Figure 1. Nutritional composition of the enteral formulas used in the study (per 100 mL)**


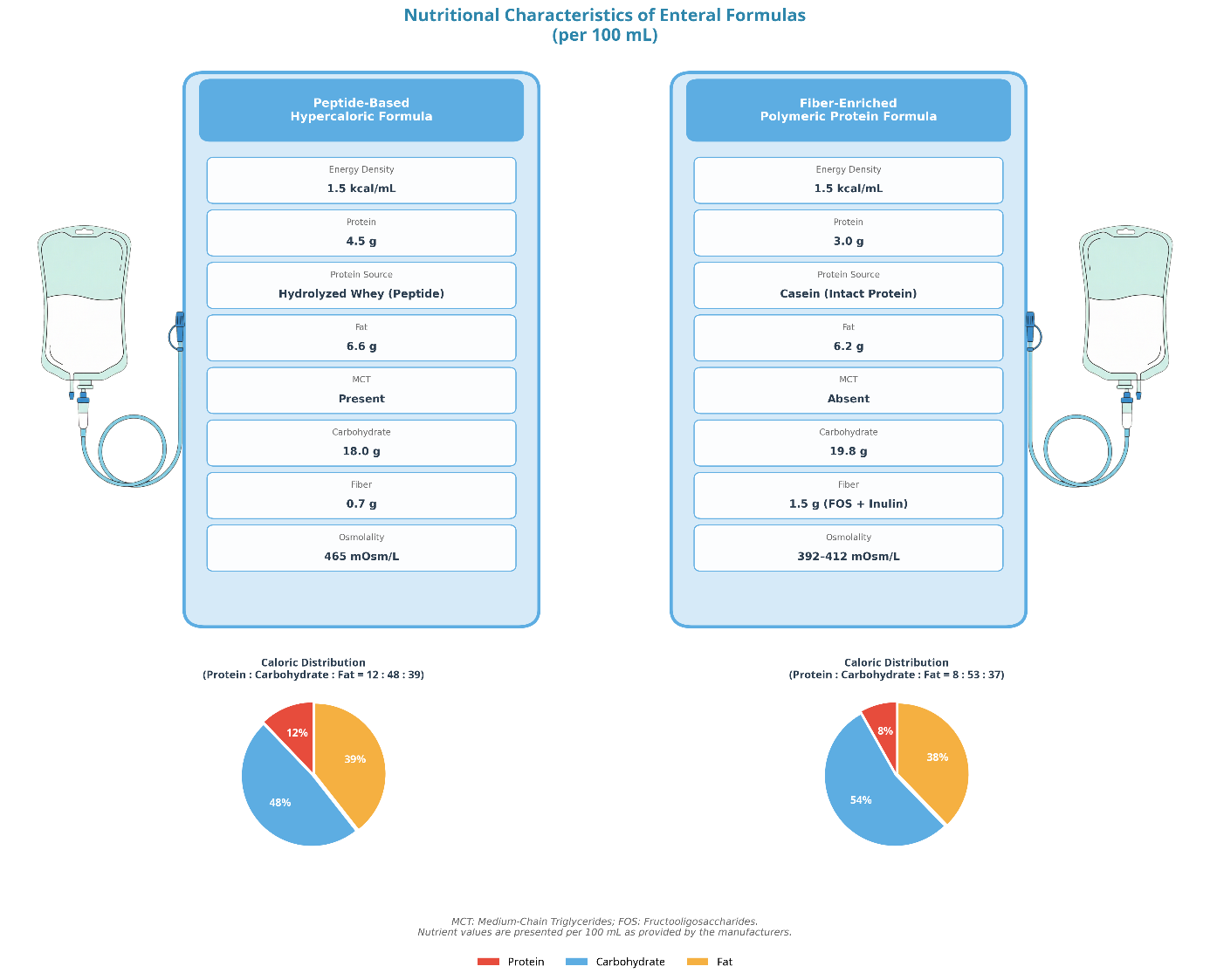


# **Supplementary Figure 2. Study flow diagram and patient selection process**


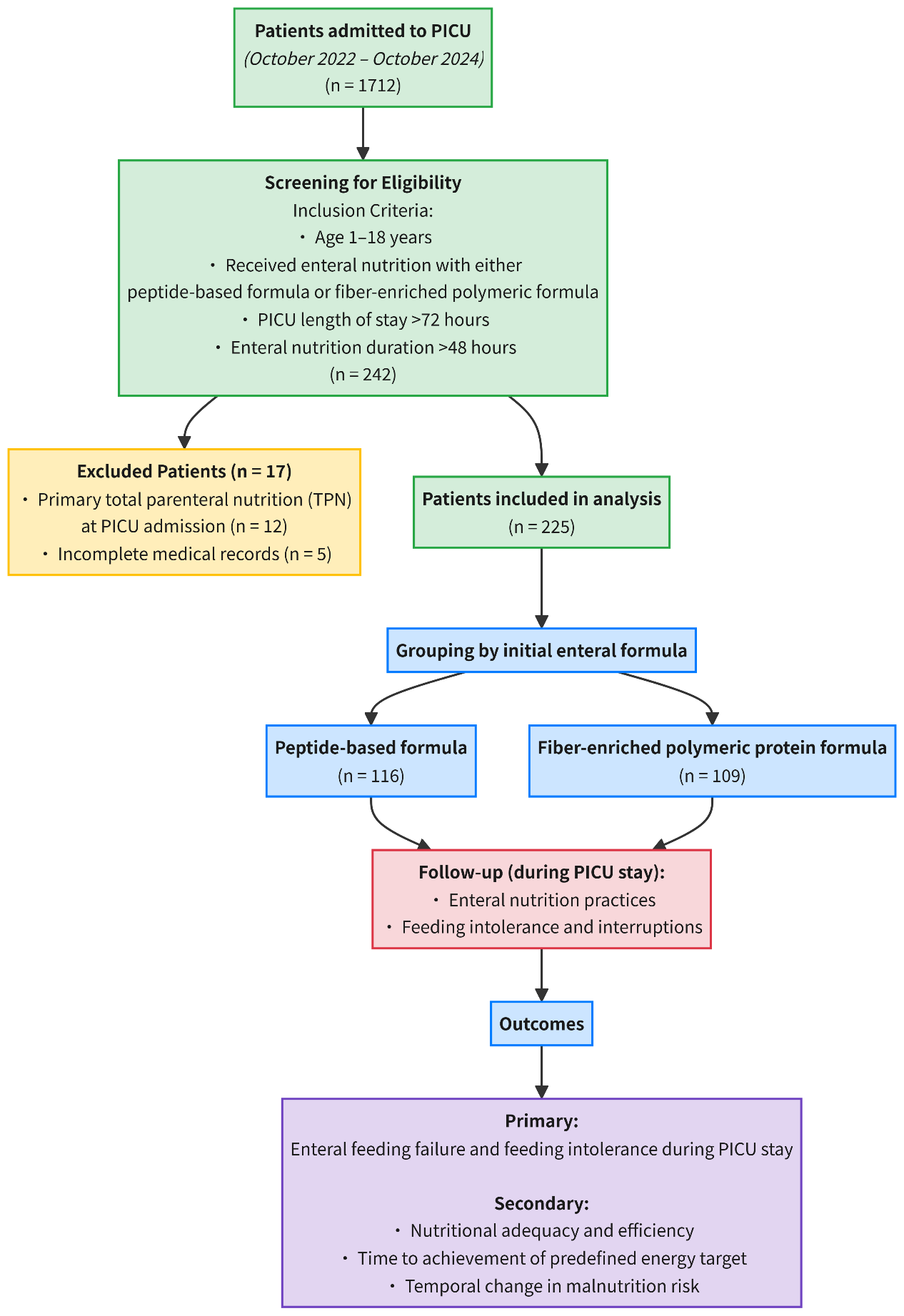


# **Supplementary Enteral Nutrition and Nutritional Monitoring Algorithm**

**1. Patient admission to the PICU**

- All patients admitted to the Pediatric Intensive Care Unit (PICU) are evaluated for eligibility for enteral nutrition in accordance with national and international pediatric critical care nutrition guidelines.
- Nutritional monitoring is initiated for all patients using standardized nutritional monitoring forms.

**2. Baseline nutritional assessment**

- On the day of PICU admission, the following anthropometric measurements are obtained:
  - Body weight
  - Height
- Measurements are performed using calibrated equipment and standardized measurement techniques.
- Nutritional status is assessed using:
  - Height-for-age Z-score
  - Weight-for-age Z-score
  - Body mass index (BMI) Z-score
- Z-scores are calculated and interpreted based on the World Health Organization (WHO) Child Growth Standards (2006).
- Anthropometric measurements are repeated at least once weekly during PICU stay.

**3. Determination of energy and protein requirements**

- Individual energy requirements are calculated using the Schofield equation.
- The nutritional goal is defined as achieving at least 66% of the calculated energy requirement by the end of the first week of enteral nutrition, in line with ASPEN recommendations.
- Protein intake is targeted at a minimum of 1.5 g/kg/day to support a positive nitrogen balance, in accordance with current pediatric critical care nutrition guidelines.

**4. Assessment of eligibility for enteral nutrition**

- Patients are evaluated for enteral nutrition initiation.
- Enteral nutrition is initiated within 24–48 hours of PICU admission in patients who are:
  - Hemodynamically stable
  - Respiratory stable
  - Without contraindications to enteral feeding

**5. Conditions requiring delay or interruption of enteral nutrition**

- Enteral nutrition is delayed or temporarily discontinued in the presence of any of the following conditions:
  - Increasing vasopressor or inotropic requirements
  - Ongoing hemodynamic instability
  - Necrotizing enterocolitis
  - Mechanical bowel obstruction
  - Active gastrointestinal bleeding
  - Intestinal ischemia
- Decisions regarding delay or interruption of enteral nutrition, including timing, duration, and clinical justification, are documented in standardized nutritional monitoring forms.

**6. Initiation and advancement of enteral feeding**

- Enteral nutrition is initiated within the first 24–48 hours of PICU admission when clinically appropriate.
- Feeding volumes are advanced gradually based on:
  - The patient’s clinical condition
  - Enteral feeding tolerance
- The timing of initiation and rate of advancement are recorded in nutritional monitoring forms.

**7. Selection of feeding route and administration method**

- The gastric route is the preferred route for enteral nutrition.
- Alternative feeding routes are selected according to existing guidelines when clinically indicated.
- In routine practice, enteral nutrition is commonly administered as:
  - Three/four hours of continuous infusion
  - Followed by three/four hours feeding break
- The final decision regarding feeding route and administration method is made by the attending clinician.
- All feeding routes, administration methods, and feeding interruptions are documented.

**8. Selection and modification of enteral formula**

- In the absence of patient-specific mandatory indications, enteral formula selection is based on clinician preference and product availability.
- In patients who tolerate enteral nutrition, the initially selected formula is continued.
- In cases of clinically significant enteral feeding intolerance:
  - A change in enteral formula and/or
  - Transition to parenteral nutrition
    is considered.
- All formula selections, changes, and associated clinical justifications are documented in nutritional monitoring forms.

**9. Daily monitoring of enteral nutrition**

- During enteral nutrition, the following parameters are monitored daily:
  - Volume of enteral nutrition administered
  - Corresponding caloric intake
  - Corresponding protein intake
  - Achievement of prescribed energy targets
  - Nutritional adequacy and nutritional efficiency
- Nutritional adequacy was assessed by achieving at least 66% of the calculated energy requirement by day 7, in conjunction with daily energy and protein intake measurements.
- Nutritional efficiency was assessed by the ability to achieve predefined caloric targets in a timely and sustained manner and was evaluated using time to first achievement of the caloric target, daily energy and protein intake during PICU stay, and the frequency of enteral nutrition interruptions, including gastric residual volume–related feeding holds.
- Enteral feeding tolerance is assessed daily based on the presence of:
  - Vomiting
  - Diarrhea (>2 mL/kg)
  - Abdominal distension
  - Constipation
  - Aspiration
  - Gastrointestinal bleeding
  - Increased gastric residual volume, defined as:
    - ≥150 mL
    - 3–5 mL/kg
    - More than half of the previous feeding volume in bolus-fed patients
    - Volumes exceeding the two-hour feeding volume in continuously fed patients
- Feeding hold is defined as a temporary interruption of enteral nutrition lasting ≤24 hours due to feeding intolerance–related clinical concerns.

**10. Documentation of nutritional interruptions and support**

- The following are systematically recorded:
  - Enteral feeding interruptions, including duration and reasons
  - Requirement for total parenteral nutrition
  - Timing of parenteral nutrition initiation
  - Total duration of parenteral nutrition
  - Enteral formula changes and clinical indications

**11. Nutritional risk assessment**

- Nutritional risk is assessed using the Pediatric Yorkhill Malnutrition Score (PYMS).
- PYMS assessment is performed and recorded at least once weekly.
- Nutritional risk monitoring is used to:
  - Evaluate feeding tolerance
  - Assess achievement of nutritional targets
  - Identify nutrition-related complications at an early stage

**12. Monitoring for refeeding syndrome**

- Refeeding syndrome is defined by the presence of:
  - Hypophosphatemia
  - Hypokalemia
  - Hypomagnesemia
    occurring during the early phase of enteral nutrition.
- Refeeding syndrome is considered a clinical entity distinct from enteral feeding intolerance.
- It is documented separately in nutritional monitoring forms.

# **Supplementary PICU Nutrition Assessment and Monitoring Form**

- **A. Patient Information**

| Patient Name: | Study/Protocol ID: | Admission Date-Time: | Admission Diagnosis: |
| --- | --- | --- | --- |
| Date of Birth / Age: | Sex: | Attending Physician: | Dietitian: |
| Height measurement method: | Weight measurement method: | Fluid restriction: | Food allergy/special diet: |
| Sedation: | IMV/NIV: | Vasoactive support: | RRT/ECMO: |

- **B. Clinical Status and Risk**

| Primary diagnosis: | Comorbidities: | PRISM III: | PELOD (max): |
| --- | --- | --- | --- |
| Hemodynamically stable? | Contraindication to EN: | NEC/obstruction/ischemia: | Active GI bleeding: |
| Escalating vasoactive support? | Shock/sepsis: | Target EN start time: | EN started? |

- **C. Anthropometric Measurements and Growth Assessment**
- Measured at admission and at least weekly. Z-scores calculated using WHO growth standards.

| Date | Day | Weight (kg) | Length/Height (cm) | BMI | WFA z | HFA z | BMI z | MUAC (cm) | Edema/Ascites |
| --- | --- | --- | --- | --- | --- | --- | --- | --- | --- |
|  |  |  |  |  |  |  |  |  |  |
|  |  |  |  |  |  |  |  |  |  |
|  |  |  |  |  |  |  |  |  |  |
|  |  |  |  |  |  |  |  |  |  |
|  |  |  |  |  |  |  |  |  |  |
|  |  |  |  |  |  |  |  |  |  |

- **D. Nutritional Risk Screening – PYMS**
- PYMS consists of four components. Reassess weekly during PICU stay.

| Component | Assessment | Score |
| --- | --- | --- |
| Recent weight loss or poor weight gain | No / Yes (specify) |  |
| Reduced intake in recent days | No / Yes (degree) |  |
| BMI percentile / z-score | Normal / Low |  |
| Effect of acute illness on nutrition | Low / Moderate / High |  |
| Total PYMS Score | Risk: Low (0) / Moderate (1) / High (2–3) / Very high (≥4) |  |

- **E. Energy and Protein Requirements**
- Energy target calculated using Schofield equation. Aim to reach ≥66% of target energy by day 7.

| Schofield BMR (kcal/day): | Stress/activity factor: | Total energy target (kcal/day): | Energy target (kcal/kg/day): |
| --- | --- | --- | --- |
| Protein target (g/day): | Protein target (g/kg/day): | Fluid limit (mL/kg/day): | Total EN volume (mL/day): |
| Planned advancement: | Day 1 %: | Day 3 %: | Day 7 ≥66%: |

- **F. Enteral Nutrition Plan**

| Start date-time: | Time from admission (hours): | Route: | |
| --- | --- | --- | --- |
| Formula name/type: | Energy density (kcal/mL): | Protein (g/100 mL): | Target rate: |
| Administration: | 3h on / 3h off: | Another: | |
| Prokinetic: | Gastric protection: | Aspiration precautions: | Post pyloric indication: |

- **G. Daily Tolerance and Interruption Monitoring**
- Monitor daily for vomiting, diarrhea, abdominal distension, constipation, aspiration, GI bleeding, feeding interruptions and reasons.

| Day | EN given? | Volume (mL) | kcal | Protein (g) | % Target | Interruption (h) | Reason | Intolerance | Notes |
| --- | --- | --- | --- | --- | --- | --- | --- | --- | --- |
| 1 |  |  |  |  |  |  |  |  |  |
| 2 |  |  |  |  |  |  |  |  |  |
| 3 |  |  |  |  |  |  |  |  |  |
| 4 |  |  |  |  |  |  |  |  |  |
| 5 |  |  |  |  |  |  |  |  |  |
| 6 |  |  |  |  |  |  |  |  |  |
| 7 |  |  |  |  |  |  |  |  |  |
| 8 |  |  |  |  |  |  |  |  |  |
| 9 |  |  |  |  |  |  |  |  |  |
| 10 |  |  |  |  |  |  |  |  |  |
| 11 |  |  |  |  |  |  |  |  |  |
| 12 |  |  |  |  |  |  |  |  |  |
| 13 |  |  |  |  |  |  |  |  |  |
| 14 |  |  |  |  |  |  |  |  |  |

- **H. Refeeding Syndrome and Metabolic Monitoring**
- Defined by hypophosphatemia, hypokalemia or hypomagnesemia after initiation of EN.

| Date | Day | Na | K | P | Mg | Glucose | Clinical signs | Intervention |
| --- | --- | --- | --- | --- | --- | --- | --- | --- |
|  |  |  |  |  |  |  |  |  |
|  |  |  |  |  |  |  |  |  |
|  |  |  |  |  |  |  |  |  |
|  |  |  |  |  |  |  |  |  |
|  |  |  |  |  |  |  |  |  |
|  |  |  |  |  |  |  |  |  |

- **I. Parenteral Nutrition / Formula Change / Special Situations**

| PN start date-time: | Indication: | Total PN days: | PN discontinuation reason: |
| --- | --- | --- | --- |
| Formula change: | Previous formula: | New formula: | Rationale: |
| Post pyloric transition: | Date: | Indication: | Complication: |
| Nutrition consult: | Dietitian note: | Physician note: | Plan: |

- **J. Weekly Review and Discharge Summary**

| Week/Date | PYMS (score, category) | Mean energy target achieved (%) | Key events/decisions |
| --- | --- | --- | --- |
|  |  |  |  |
|  |  |  |  |
|  |  |  |  |
|  |  |  |  |
|  |  |  |  |
|  |  |  |  |
|  |  |  |  |
|  |  |  |  |

- Discharge / transfer nutrition plan: _________________________________________________
- Signature – Physician: __________ Dietitian: __________ Nurse: __________

# **Supplementary Statistical Methods**

No formal sample size or power calculation was performed, as this study was designed as a retrospective observational analysis. The study cohort comprised all patients admitted to the Pediatric Intensive Care Unit (PICU) during the study period who met the predefined inclusion criteria. Data were retrospectively obtained from standardized electronic hospital records. To ensure data comparability, all variables were collected using identical data sources, definitions, and measurement methods across both enteral formula groups.

The Pediatric Yorkhill Malnutrition Score (PYMS) was used to assess nutritional risk. PYMS is a validated screening tool for pediatric populations and consists of four components: recent weight loss or inadequate weight gain, reduced food intake, body mass index percentile, and the potential impact of acute illness on nutritional status. Total PYMS scores range from 0 to 7 points, and patients were categorized into four nutritional risk groups: low risk (0 points), moderate risk (1 point), high risk (2–3 points), and very high risk (≥4 points). PYMS assessments were performed once weekly during the PICU stay and were used for longitudinal evaluation of nutritional risk. Results are presented graphically using bar charts. For patients who underwent a formula change, only PYMS scores recorded prior to the change were included in the longitudinal analysis to avoid confounding the effect of the initial formula type.

Continuous variables were summarized as medians with interquartile ranges (IQR), and categorical variables were expressed as frequencies and percentages. Nutritional adequacy was evaluated using predefined clinically relevant thresholds, including achievement of at least 66% of the prescribed energy target by day 7 of enteral nutrition. Time-to-event outcomes, such as time to first achievement of caloric targets, were analyzed using Kaplan–Meier survival methods, with between-group comparisons performed using the log-rank test.

To identify factors associated with enteral feeding failure, variables demonstrating a p-value <0.1 in initial bivariate comparisons were entered into univariable logistic regression analyses. Variables with p <0.1 in univariable analyses were subsequently considered candidates for multivariable logistic regression models. Results were reported as adjusted odds ratios (aORs) with corresponding 95% confidence intervals (CIs). Model assumptions were assessed using standard diagnostic methods, including evaluation of collinearity among covariates.

Longitudinal changes in nutritional, clinical, and laboratory outcomes during the PICU stay were analyzed using generalized estimating equations (GEE) for repeated measurements. Patient identification number was specified as the subject variable, and follow-up day was specified as the within-subject variable, using an autoregressive correlation structure. Enteral formula group (peptide-based vs. fiber-enriched polymeric) and time were included as main effects, with interaction terms used to evaluate differential temporal trends between groups. Continuous outcomes were analyzed assuming a normal distribution with an identity link function, and results were reported as β-coefficients with 95% confidence intervals. Binary outcomes were analyzed using a binomial distribution with a logit link function, and results were reported as odds ratios with 95% confidence intervals. Robust variance estimators were applied to account for within-subject correlation.

Patients with missing data for variables required in specific analyses were excluded from those analyses using a complete-case approach. Given the retrospective nature of the study and the limited proportion of missing data for key variables, no data imputation was performed. Statistical significance was defined as a two-sided p-value <0.05. All statistical analyses were conducted using IBM SPSS Statistics software (Version 26.0; IBM Corp., Armonk, NY, USA). Results are presented using effect estimates and corresponding confidence intervals to facilitate clinical interpretation and to reduce overreliance on null hypothesis significance testing. Detailed descriptions of statistical methods, model specifications, and analytic decisions are provided to support transparency and reproducibility.

# **Supplementary Figure 3. Kaplan–Meier Analysis of Time to Caloric Goal Achievement by Enteral Formula Type**


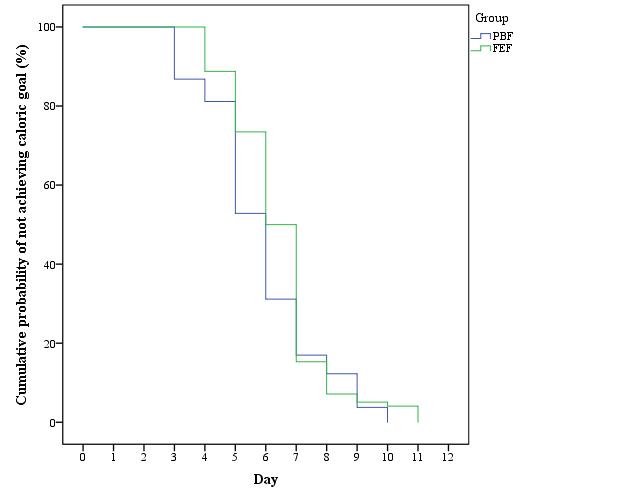


Kaplan–Meier curves depicting time to achieve caloric goals in patients receiving peptide-based formula (PBF) versus fiber-enriched formula (FEF). Patients in the PBF group achieved caloric targets earlier, as reflected by a more rapid decline in the cumulative probability of not achieving caloric goals. The mean time to caloric goal attainment was 5.85 days (95% CI: 5.50–6.20) for PBF and 6.44 days (95% CI: 6.13–6.75) for FEF, (log-rank test, p = 0.035).

# **Supplementary Table 1: Feeding Failure Comparison Table**

| **Variable** | **Feeding failure (n = 81)** | **No feeding failure (n = 144)** | **p value** |
| --- | --- | --- | --- |
| Age, months, median (IQR) | 88.0 (53.0-124.0) | 106.5 (74.8-138.0) | **0.020** |
| Sex, male, n (%) | 42 (51.9%) | 77 (53.5%) | 0.925 |
| Weight, kg, median (IQR) | 16.9 (10.4-22.3) | 18.2 (13.1-24.4) | 0.056 |
| BMI z-score, median (IQR) | -0.6 (-1.8-0.3) | -0.7 (-3.1-0.2) | 0.570 |
| Primary diagnosis category, n (%) |  |  |  |
| - Respiratory | 20 (24.7%) | 49 (34.0%) | 0.244 |
| - Neurologic | 7 (8.6%) | 17 (11.8%) |  |
| - Sepsis / infection | 14 (17.3%) | 14 (9.7%) |  |
| - Cardiac | 10 (12.3%) | 15 (10.4%) |  |
| - Postoperative | 7 (8.6%) | 19 (13.2%) |  |
| - Other | 11 (13.6%) | 3 (2.1%) |  |
| Comorbidity present, n (%) | 44 (54.3%) | 53 (36.8%) | **0.016** |
| PRISM III score, median (IQR) | 14.0 (10.0-24.0) | 12.0 (6.0-18.0) | **0.008** |
| PELOD score, (max) median (IQR) | 16.0 (14.0-18.0) | 16.0 (14.0-18.0) | 0.606 |
| IMV, n (%) | 28 (34.6%) | 45 (31.2%) | 0.717 |
| Vasoactive support, n (%) | 11 (13.6%) | 17 (11.8%) | 0.860 |
| Renal replacement therapy, n (%) | 6 (7.4%) | 7 (4.9%) | 0.553 |
| ECMO, n (%) | 2 (2.5%) | 1 (0.7%) | 0.295 |
| Time to enteral nutrition initiation, days, median (IQR) | 1.5 (1.0-2.5) | 1.5 (1.0-2.5) | 0.615 |
| Enteral feeding route, n (%) |  |  |  |
| - Nasogastric | 64 (79.0%) | 108 (75.0%) | 0.615 |
| - Orogastric | 3 (3.7%) | 8 (5.6%) |  |
| - Gastrostomy | 12 (14.8%) | 25 (17.4%) |  |
| - Postpiloric | 2 (2.5%) | 3 (2.1%) |  |
| **Baseline laboratory values (Day 1)** |  |  |  |
| Albumin, g/dL | 3.2 (2.9-3.4) | 3.2 (2.9-3.4) | 0.747 |
| Sodium, mmol/L | 138.0 (135.0-140.0) | 137.0 (136.0-139.0) | 0.214 |
| Potassium, mmol/L | 4.3 (3.9-4.5) | 4.3 (4.0-4.6) | 0.323 |
| Phosphate, mg/dL | 3.6 (3.4-3.9) | 3.6 (3.2-3.9) | 0.407 |
| Urea, mg/dL | 24.0 (19.0-30.0) | 25.0 (19.8-30.0) | 0.816 |
| Glucose, mg/dL | 110.0 (95.0-120.0) | 110.0 (100.8-122.5) | 0.350 |
| Peptide-based hypercaloric formula, n (%) | 34 (42.0%) | 82 (56.9%) | **0.044** |
| Fiber-enriched polymeric formula, n (%) | 47 (58.0%) | 62 (43.1%) |  |

BMI, body mass index; PRISM III, Pediatric Risk of Mortality III score; PELOD, Pediatric Logistic Organ Dysfunction score; IMV, invasive mechanical ventilation; ECMO, extracorporeal membrane oxygenation; IQR, interquartile range.
